# Cerebral Metastasis in a Fatal Adrenocortical Carcinoma: A Rare Presentation of an Aggressive Tumor

**DOI:** 10.3390/diagnostics16081143

**Published:** 2026-04-11

**Authors:** Ach Taieb, Amira Yanes, Rihab Ben Fredj, Majdouline Barkache, Oumaima Zarrouk, Wiem Saafi, Imen Halloul, Hamza El Fekih, Zeineb Lajmi, Yasmine Ben Romdhane, Ghada Saad, Yosra Hasni

**Affiliations:** 1 Faculty of Medicine of Sousse, University of Sousse, Sousse 4000, Tunisia; amira.yanes@famso.u-sousse.tn (A.Y.); rihab.ben.fredj@hotmail.com (R.B.F.); majdouline.barkache@famso.u-sousse.tn (M.B.); oumaymazarrouk59@gmail.com (O.Z.); wiem.saafi@gmail.com (W.S.); imen.halloul22@gmail.com (I.H.); elfekihamza@gmail.com (H.E.F.); zeineb.lajmi@famso.u-sousse.tn (Z.L.); yasmine.benromdhane@fmaso.u-sousse.tn (Y.B.R.); ghada.saad6587@gmail.com (G.S.); y.hasni@gmail.com (Y.H.); 2 Department of Endocrinology, University Hospital of Farhat Hached Sousse, Sousse 4031, Tunisia; 3 Laboratory of Exercise Physiology and Pathophysiology, L.R.19ES09, Sousse 4054, Tunisia; 4 Department of Neurosurgery, University Hospital of Sahloul Sousse, Sousse 4054, Tunisia; 5 Department of Pathology, University Hospital of Sahloul Sousse, Sousse 4054, Tunisia

**Keywords:** adrenocortical carcinomas, cancer, metastasis, cortisol

## Abstract

Adrenocortical carcinomas (ACCs) are rare, aggressive tumors often discovered incidentally. These malignancies may present with abnormal hormone secretion or, as in some cases, as non-functioning masses causing discomfort. We present a case of brain metastasis in a patient with a giant ACC. A 50-year-old man presented with headache and dizziness. A computed tomography (CT) scan showed an intracranial lesion within the parenchyma measuring 73*60*46 mm with left internal temporal involvement, abundant vasogenic edema and compressing the lateral left ventricle. Further imaging investigations identified a large necrotic tissue mass measuring 15 cm, located on both sides of the right diaphragmatic dome, in the middle posterior region. Hormonal workup was conducted and excluded a functional adrenal tumor. A CT-guided biopsy was performed, confirming ACC. Despite medical management, the patient’s condition deteriorated rapidly, with the cerebral metastasis proving fatal. This case underscores the challenges posed by advanced ACC, particularly when associated with atypical metastatic sites. Giant ACC, though rare, presents significant diagnostic and therapeutic challenges. Surgical excision with appropriate oncologic management can lead to favorable outcomes. This report contributes to the limited literature on cerebral metastases in ACC, aiming to enhance awareness among clinicians managing this rare entity.

**Figure 1:**
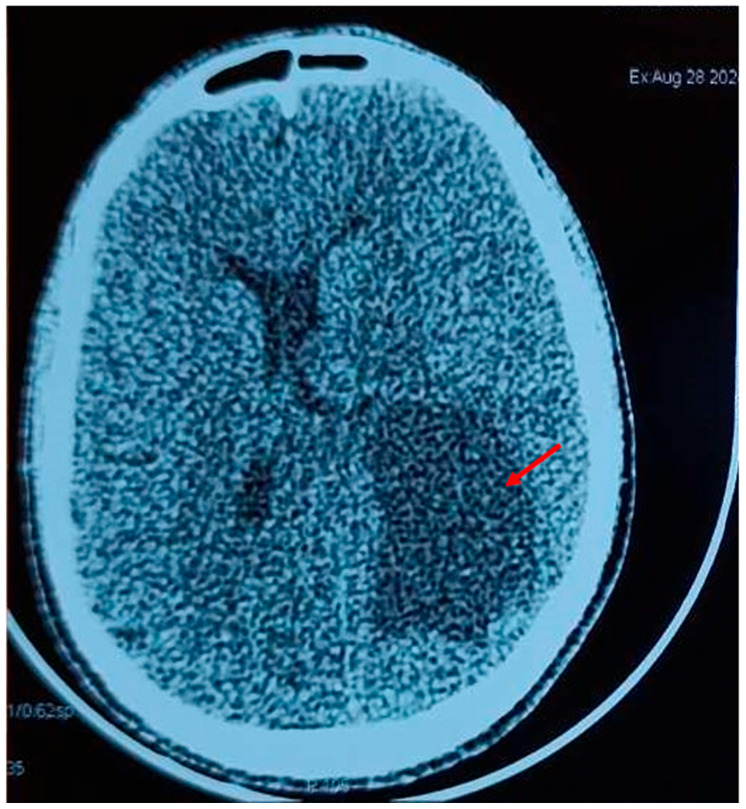
A 50-year-old man with no significant medical history presented with headaches and dizziness. Non-contrast axial brain CT scan demonstrating a large, heterogeneous parenchymal lesion (73 × 60 × 46 mm) in the left internal temporal region, with irregular margins, surrounding vasogenic edema, and mass effect compressing the left lateral ventricle.

**Figure 2:**
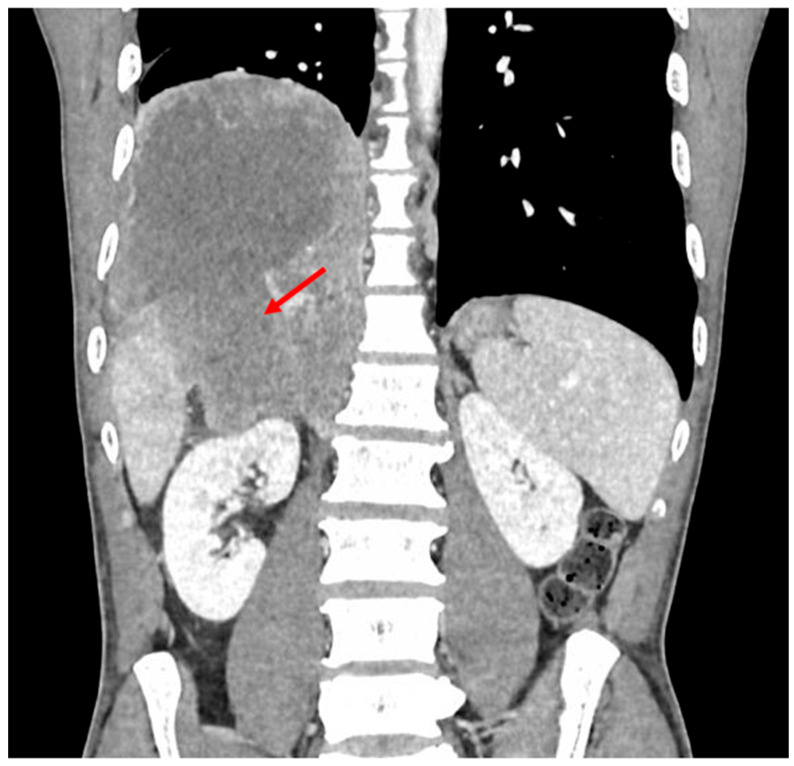
Contrast-enhanced abdominal CT scan (axial view) showing a large (15 cm) heterogeneous, necrotic adrenal mass with irregular contours, located bilaterally beneath the right diaphragmatic dome in the middle posterior region.

**Figure 3:**
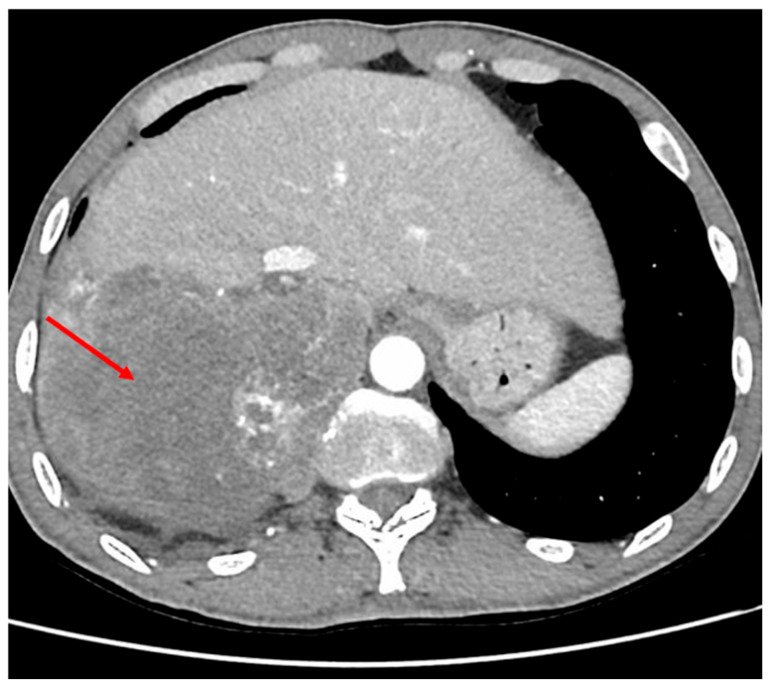
Coronal contrast-enhanced abdominal CT scan demonstrating the adrenal mass displacing the liver and inferior vena cava. No evidence of pulmonary, mediastinal, pleural, or bone metastases. Hormonal workup was conducted to exclude functional adrenal tumors [1]. Serum cortisol, 24 h urinary free cortisol, and ACTH levels were normal, ruling out Cushing’s syndrome. Plasma and urinary metanephrines were within normal limits, excluding pheochromocytoma. The aldosterone-to-renin ratio was also normal, eliminating hyperaldosteronism. Serum dehydroepiandrosterone sulfate, androstenedione, testosterone, and 17-hydroxyprogesterone levels were all within normal reference ranges, further supporting the non-functional status of the tumor.

**Figure 4:**
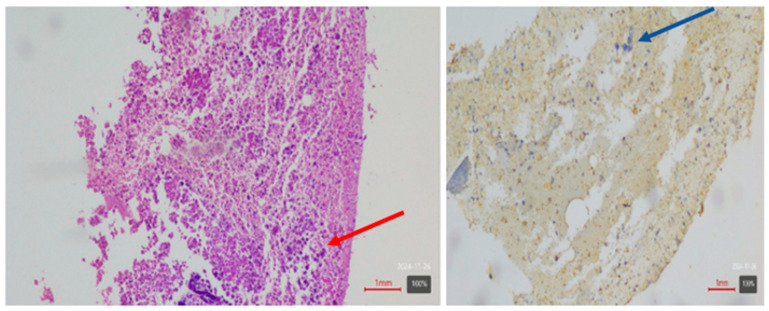
Histopathological examination of the CT-guided biopsy specimen revealed a malignant adrenal cortical tumor. The Weiss score was 7 (including high nuclear grade, >5 mitoses per 50 high-power fields, atypical mitoses, diffuse architecture, and tumor necrosis), consistent with adrenocortical carcinoma. Hematoxylin and eosin stain, ×20 magnification, showed diffuse architecture, high nuclear grade, and atypical mitoses (red arrow). Immunohistochemistry showed diffuse cytoplasmic positivity for inhibin (×20), supporting adrenocortical origin (blue arrow). A surgical management involving adrenalectomy was scheduled, along with chemotherapy treatment based on mitotane. Unfortunately, the patient’s condition rapidly deteriorated over the course of two weeks, leading to fatal brain herniation. The clinical presentation of ACC varies significantly depending on the tumor’s size and its functional status regarding hormone production [2]. Smaller, non-functioning adrenal tumors are frequently found incidentally during imaging studies performed for other reasons, hence called incidentalomas [3,4]. Less than 2% of incidentalomas under 4 cm in size are primary adrenal carcinomas. However, the risk of malignancy increases substantially for masses larger than 6 cm, where 25% of these adrenal masses are malignant [5,6]. Surgical resection remains the primary treatment for adrenal cortical carcinoma. Radical excision with clear margins (R0 resection) is the most important prognostic factor, as it significantly impacts survival outcomes [7]. For adrenal tumors larger than 6 cm or those showing signs of malignancy, open adrenalectomy is the preferred approach. In cases where laparoscopic techniques are applied, they are typically reserved for smaller tumors [8]. Mitotane, an adrenolytic drug, is the mainstay of systemic therapy for ACC, particularly in advanced or recurrent cases. In addition to mitotane, combination chemotherapy regimens such as EDP-M (etoposide, doxorubicin, cisplatin plus mitotane) or streptozotocin plus mitotane are often used [9,10]. The most frequent symptoms associated with brain metastases from ACC in the published series [5] were headache, focal neurological deficits, and altered mental status. Brain metastases commonly occur in patients with lung cancer, breast cancer and melanoma, but are uncommon in those with ACC. This case is particularly noteworthy for the discovery of a large, symptomatic brain metastasis preceding the identification of the primary adrenocortical carcinoma, an unusual presentation that underscores the aggressive nature of this tumor and the importance of considering metastatic workup even in the absence of prior adrenal diagnosis.

## Data Availability

Data are available from the authors upon request.

## References

[1] Sharma E., Dahal S., Sharma P., Bhandari A., Gupta V., Amgai B., Dahal S. (2018). The characteristics and trends in adrenocortical carcinoma: A United States popu-lation based study. J. Clin. Med. Res..

[2] Gasmi A., Taieb A., Hattab F., Ghachem A., Slama N.B.H., Halloul I., Saafi W., Marzouk H., Elfekih H., Saad G. (2025). Characterization of Unilateral Adrenal Incidentalomas: Hormonal Analysis, Computed Tomography, and Magnetic Resonance Imaging Correlation. Cureus.

[3] Lerario A.M., Moraitis A., Hammer G.D. (2014). Genetics and epigenetics of adrenocortical tumors. Mol. Cell. Endocrinol..

[4] Gatta-Cherifi B., Chabre O., Murat A., Niccoli P., Cardot-Bauters C., Rohmer V., Young J., Delemer B., Du Boullay H., Verger M.F. (2012). Adrenal involvement in MEN1. Analysis of 715 cases from the Groupe d’etude des Tumeurs Endocrines database. Eur. J. Endocrinol..

[5] Thampi A., Shah E., Elshimy G., Correa R. (2020). Adrenocortical carcinoma: A literature review. Transl. Cancer Res..

[6] Shariq O.A., McKenzie T.J. (2021). Adrenocortical carcinoma: Current state of the art, ongoing controversies, and future directions in diagnosis and treatment. Ther. Adv. Chronic Dis..

[7] Fassnacht M., Assie G., Baudin E., Eisenhofer G., de la Fouchardiere C., Haak H., de Krijger R., Porpiglia F., Terzolo M., Berruti A. (2020). Adrenocortical carcinomas and malignant phaeochromocytomas: ESMO-EURACAN Clinical Practice Guidelines for diagnosis, treatment and follow-up. Ann. Oncol. Off. J. Eur. Soc. Med. Oncol..

[8] Fassnacht M., Dekkers O.M., Else T., Baudin E., Berruti A., de Krijger R.R., Haak H.R., Mihai R., Assie G., Terzolo M. (2018). European Society of Endocrinology Clinical Practice Guidelines on the management of adrenocortical carcinoma in adults, in collaboration with the European Network for the Study of Adrenal Tumors. Eur. J. Endocrinol..

[9] Chukkalore D., MacDougall K., Master V., Bilen M.A., Nazha B. (2024). Adrenocortical Carcinomas: Molecular Patho-genesis, Treatment Options, and Emerging Immunotherapy and Targeted Therapy Approaches. Oncologist.

[10] Szyszka P., Grossman A.B., Diaz-Cano S., Sworczak K., Dworakowska D. (2016). Molecular pathways of human adrenocortical carcinoma—Translating cell signalling knowledge into diagnostic and treatment options. Endokrynol. Pol..

